# Delirium in the ICU: how much do we know? A narrative review

**DOI:** 10.1080/07853890.2024.2405072

**Published:** 2024-09-23

**Authors:** Si Bo Liu, Hong Yu Wu, Mei Li Duan, Rong Li Yang, Chen Hua Ji, Jin Jie Liu, Hongtao Zhao

**Affiliations:** a Intensive Care Unit, Dalian Municipal Central Hospital Affiliated Dalian University of Technology, Dalian, China; b Intensive Care Unit, Capital Medical University Affiliated Beijing Friendship Hospital, Beijing, China; c General Medicine Ward, Dalian Municipal Central Hospital Affiliated Dalian University of Technology, Dalian, China

**Keywords:** Delirium, intensive care unit, biomarkers, diagnosis, treatment

## Abstract

Delirium in critical ill patients is a complex and common neurological syndrome in the intensive care unit (ICU) that is caused by a range of structural or functional abnormalities. ICU Delirium is associated with reduced compliance, prolonged hospital stays, greater use or delayed withdrawal of sedatives, higher rates and durations of mechanical ventilation, and higher rates of mortality. The aetiology and pathogenesis of ICU delirium are unclear, and the lack of better prediction, prevention, and treatment measures leads to a non-standardized control of delirium. By searching the relevant literature, we aim in this narrative review to describe progress in the pathogenesis, predictive biomarkers, diagnosis, and treatment of ICU delirium.

## 1. Foreword

Delirium is a common acute disturbance of mental status, with an incidence rate of 20–50% in the intensive care unit (ICU) [1] and up to 80% in patients on mechanical ventilation [2]. Once delirium occurs, patient compliance is severely compromised. Severity of illness, prolonged sedation and mechanical ventilation are independent risk factors associated with in-hospital mortality in patients with delirium [2,3]. Although local recommendations and international guidelines have been published [4,5], they are often not followed [6,7]. A worldwide online survey disclosed that although respondents acknowledged the need for delirium monitoring, more than 58% of them did not use specific tools to monitor delirium [7]. For a long time, insufficient attention has been given to the normative diagnosis and management of delirium in the ICU, in both adult and paediatric patients [6–8]. Second, the tools used for diagnosis and treatment evaluation differ between countries, regions, hospitals, and even departments [9–11]. In recent years, with the continued development of diagnostic criteria for delirium and the gradual exploration of treatment interventions, clinicians have gradually paid more attention to delirium in the ICU [5,12]. This narrative review summarizes the pathogenesis, predictive biomarkers, diagnostic criteria, prevention and treatment measures, and potential therapeutic targets of ICU delirium by systematically reviewing recent works to provide a theoretical basis for further research and clinical practice.

## 2. Pathogenesis and biomarkers

The pathophysiology of delirium is complex, involving multiple interactions between aetiologies and precipitating factors that are still poorly explored. Understanding the mechanisms will help to predict the risk of delirium as early as possible and will encourage clinicians to implement interventions to reduce the incidence of delirium.

### 2.1. Neurological disease and imaging biomarkers

Aging and neurological diseases are important susceptibility factors for delirium. Patients with delirium observed more brain atrophy, white matter lesions, and ischemic and hypoxic vascular lesions, as indicated by different MRI sequences [13–15]. About 54.9% of patients with delirium have brain lesions, and 16.1% have brain atrophy on MRI; these abnormal MRI findings are associated with reduced C5a and IC3b levels and increased tau levels [15]. Decreased arousal network activity and an imbalance in cortico-subcortical hemispheric connectivity have also been associated with the onset of delirium in patients with MRI abnormalities [16]. Compared with other biomarkers, preexisting brain atrophy and brain lesions before delirium onset may help to rapidly identify individuals at high risk of delirium. However, the relationship between these imaging changes and the development of delirium remains unclear. The value of imaging findings in predicting ICU delirium needs further study.

### 2.2. Metabolic disorders and humoral biomarkers

**Figure 1.**
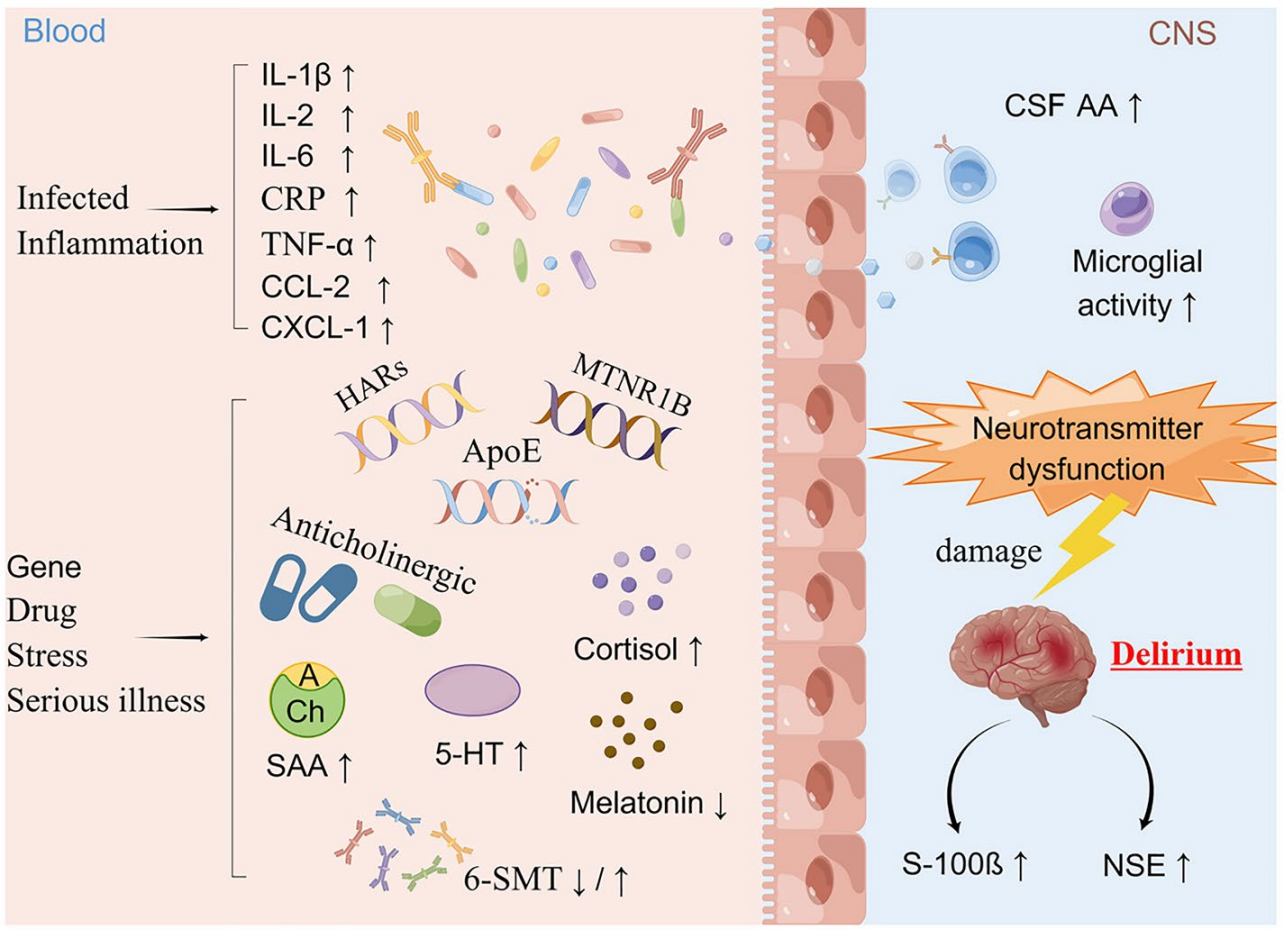
Potential pathophysiology of delirium. IL-1β, interleukin 1β; IL-2, interleukin 2; IL-6, interleukin 6; CRP, C-reactive protein; TNF-α, tumour necrosis factor α; CCL-2, C-C chemokine ligand 2; CXCL-1, chemokine C-X-C motif ligand 1; HARs, human accelerated regions; MTNR1B, melatonin receptor 1B gene; Apo-E, apolipoprotein E; SAA, serum anticholinergic activity; CSF AA, cerebrospinal fluid anticholinergic activity; 5-HT, serotonin; 6-SMT, 6-hydroxymelatonin sulfate; S-100β, S100 calbindin B; NSE, neuron-specific enolase. Drawn with Figdraw.

Metabolic disorders contribute to delirium onset or act as mediators between drugs [17,18], systemic inflammation [19,20], sleep deprivation [21], physical restraints [21,22], and other changes in the surrounding environment and acute abnormalities of the nervous system (Figure 1).

Neurotransmitter imbalances, especially of acetylcholine, are among the most reported risk biomarkers for delirium [23]. In patients with respiratory failure or shock, a higher daily plasma acetylcholinesterase was associated with an increased risk of delirium compared with a normal mental status on the same day [24]. In patients with sepsis, approximately 90% exhibit statistically significant decreases in acetylcholinesterase activity over a period of at least 5 consecutive days from baseline [25], while longitudinal changes were observed only in patients with suspected septic-associated encephalopathy and could be used to diagnose septic-associated encephalopathy in patients with delirious symptoms [25]. In addition, postoperative plasma gamma-aminobutyric acid was independently associated with delirium in critical illness patients [26]. Although neurotransmitter testing has not been included in the scope of laboratory testing, making bedside testing difficult to achieve at present. However, it has been gradually incorporated into disease screening in hospitals, and may become a bedside monitoring biomarker in the future.

Systemic inflammation is another common risk factor for delirium in critical illness patients. Inflammatory mediators may cross the blood–brain barrier, leading to functional or structural impairment in the central nervous system [27–29]. Significantly higher serum neutrophil–lymphocyte ratios were found in elderly patients with critical illness with delirium than in those with a normal mental status [30]. A prospective study enrolled 78 patients admitted over 24 h in the ICU and collected blood samples within 12 h of enrolment. The results showed that soluble tumour necrosis factor (TNF) receptor-1 and -2, adiponectin, and interleukin (IL)-1β levels were higher in patients with delirium occurrence during the first 72 h of ICU admission [31]. IL-6, IL-8, IL-10, IL-18, TNF-α, and chemokines (CCL2, CCL3, CXCL1, and CXCL10) have also been reported to be elevated in ICU delirium patients and are associated with delirium severity [19,31,32]. Therefore, inflammatory markers and cytokines are potential biomarkers for the prediction of ICU delirium.

Other metabolic biomarkers have also been related to ICU delirium. One of the most important stress hormones is cortisol. Under stress conditions, increased adrenal axis reactivity, excessive secretion of cortisol, and the use of glucocorticoids can cause cognitive and mental disorders such as mood and memory disturbances [17,33]. Patients with septic delirium had significantly higher plasma cortisol levels than patients without delirium [34].

No delirium-specific serum markers have been identified. Analyses of relevant humoral metabolic biomarkers after ICU admission and before the onset of delirium are useful for identifying the prevalence of delirium, so these may become therapeutic targets for reducing the risk of delirium, especially the levels of neurotransmitters and inflammatory mediators.

## 3. Tools for screening, diagnosis, and therapeutic effect assessment

Delirium can be divided into hyperactive, hypoactive, and mixed delirium according to the characteristics of the symptoms [35]. In the clinic, hyperactive delirium and mixed delirium are more easily identified, while hypoactive delirium is often overlooked due to the lower state of consciousness. Many methods for screening and diagnosing delirium are often used in the clinic and in research [9,11,36–44] (Table 1). The Statistical Diagnostic Manual of Mental Disorders (DSM) published by the American Psychiatric Association and the International Classification of Diseases are the gold standard for diagnosing mental disorders worldwide [45] and are the strictest criteria based on symptoms and aetiology; this standard mostly needs to be applied by neurologists and psychiatrists. The DRS-R-98 assessment method is more rigorous and detailed, and can be used to distinguish between hyperactive and hypoactive delirium, with a sensitivity of 92% and specificity of 95% [41], though it still has shortcomings and suffers from too little evidence in ICU patients [46]. As delirium assessment in the ICU is carried out mostly by bedside nurses, a simple and fast score based on symptoms is more applicable for screening. Compared with CAM [36,47], the Confusion Assessment Method for the Diagnosis of Delirium in the ICU (CAM-ICU) and Intensive Care Delirium Screening Checklist (ICDSC) are more often used for the diagnosis of critical illness and are recommended by ICU guidelines [5,8]. CAM-ICU is superior in ruling out patients without ICU delirium and in detecting delirium in patients with ventilation and has higher summary specificity than ICDSC [48,49]. In randomized controlled trials, CAM-ICU is the most used tool to evaluate the effects of pharmacological and nonpharmacological therapies on the primary outcome of delirium incidence in randomized controlled trials (RCTs) [50–52].

**Table 1.** Common diagnostic methods for ICU delirium in the clinic and related studies.

Type of tools	Methods	Abbreviations
Diagnosis	Diagnostic and statistical manual of mental disorders-5	DSM-5
Screening	Confusion assessment method for the intensive care unit	CAM-ICU
	Intensive care delirium screening checklist	ICDSC
	The confusion assessment method	CAM
Delirium severity	Confusion assessment method-severity	CAM-S
	Confusion assessment method for the intensive care unit-7	CAM-ICU-7
	Delirium rating scale-revised-98	DRS-R-98
	Memorial delirium assessment score	MDAS
Sedative level	Richmond agitation and sedation scale	RASS
	Sedation-agitation scale	SAS
	Ramsay sedation scale	Ramsay
	Observer’s assessment of alertness/sedation scale (OAA/S)	(OAA/S)

ICU, intensive care unit.

For adult ICU patients with hyperactive delirium, sedatives such as continuous IV infusions of dexmedetomidine rather than benzodiazepine infusions are preferred by clinicians according to guideline recommendation [53]. These patients may need to be evaluated for both sedation level and delirium control. Two tools are most often used to assess the effect of sedation: the Richmond Agitation and Sedation Scale (RASS) [54] and the Sedation-Agitation Scale (SAS), which are also recommended by ICU guidelines [4,5]. RASS is supported by the most evidence from RCTs [55–57]. CAM-ICU combines the degree of sedation and can be assessed at the same time as RASS [10]. Sedatives can be titrated to maintain either light or deep sedation. Multiple studies support lighter sedation levels in adult ICU patients to improve outcomes, including a shortened duration of mechanical ventilation, a shorter hospital stay, and less long-term cognitive dysfunction [53], and it was beneficial for delirium screening and monitoring.

Based on the above evidence, CAM-ICU is the most appropriate tool for screening and monitoring ICU delirium, especially for patients under sedation.

## 4. Prevention and treatment

There is a comprehensive management system of nonpharmacological and pharmacological interventions for the prevention and treatment of delirium in the ICU. Intervention of primary disease and the reduction of medical triggers, such as inflammatory responses [27–29], abnormal energy metabolism caused by hypoxia and ion disturbance [58,59], an uncomfortable environment [21], and drugs, such as the intraoperative application of dopamine and analgesic ketamine [60], sedative midazolam [61], and benzodiazepines [62,63] (Table 2), are important measures for preventing delirium.

**Table 2.** Drugs often used in the ICU that increase the risk of delirium.

Type of drugs	Measures to avoid delirium onset or recurrent if must be used	Typical drugs
Corticosteroids	Low-dose	Glucocorticoids
Benzodiazepine, benzodiazepine receptor antagonist	Low-dose, avoid sudden withdrawal, and weaned over several days	Alprazolam, Lorazepam, Midazolam, flumazenil
Opioids, opioid antagonist, naloxone, or mixed agonist/antagonists	Low-dose, avoid sudden withdrawal, and weaned over several days	Sufentanil, Nalbuphine
Anaesthetic	Low-dose, avoid sudden withdrawal, and weaned over several days	Propofol, Ketamine
Anticholinergic drugs	Low-dose	Atropine

Nonpharmacological interventions, which mainly include control of the environment (avoiding noise, confusing stimuli, continuous light stimulation, sleep deprivation, maintaining circadian rhythm, etc.), cognitive functional rehabilitation training, family nursing knowledge training, and music training, are the cornerstone of delirium prevention/management and are recommended first line by all guidelines published to date, both adult and paediatric [4,5,64–70]. If the effectiveness of nonpharmacological interventions I limited, pharmacologic treatment with antipsychotics (or other agents) should be limited to those with severe symptoms and/or those with nonpharmacologic interventions that have failed. The treatment of elderly critically ill patients is often complicated by the presence of multiple diseases, an attention should be given to controlling the types or standard use of drugs (Table 2) in high-risk patients. Reducing or avoiding the use of analgesic, delirium-active sedatives such as benzodiazepines, psychotropic and hormonal drugs during procedures and after ICU admission can reduce the onset or recurrence of delirium [18,71,72], which need a cooperated attention of anaesthesiologists, surgeons, and practicing intensivists, as an exposure to these drugs such as benzodiazepine has been reported to be correlated with an increased risk of delirium [73,74]. If drug application cannot be avoided, low-dose, serological drug concentration testing or the avoidance of sudden withdrawal may be important preventive measures [18].

**Figure 2.**
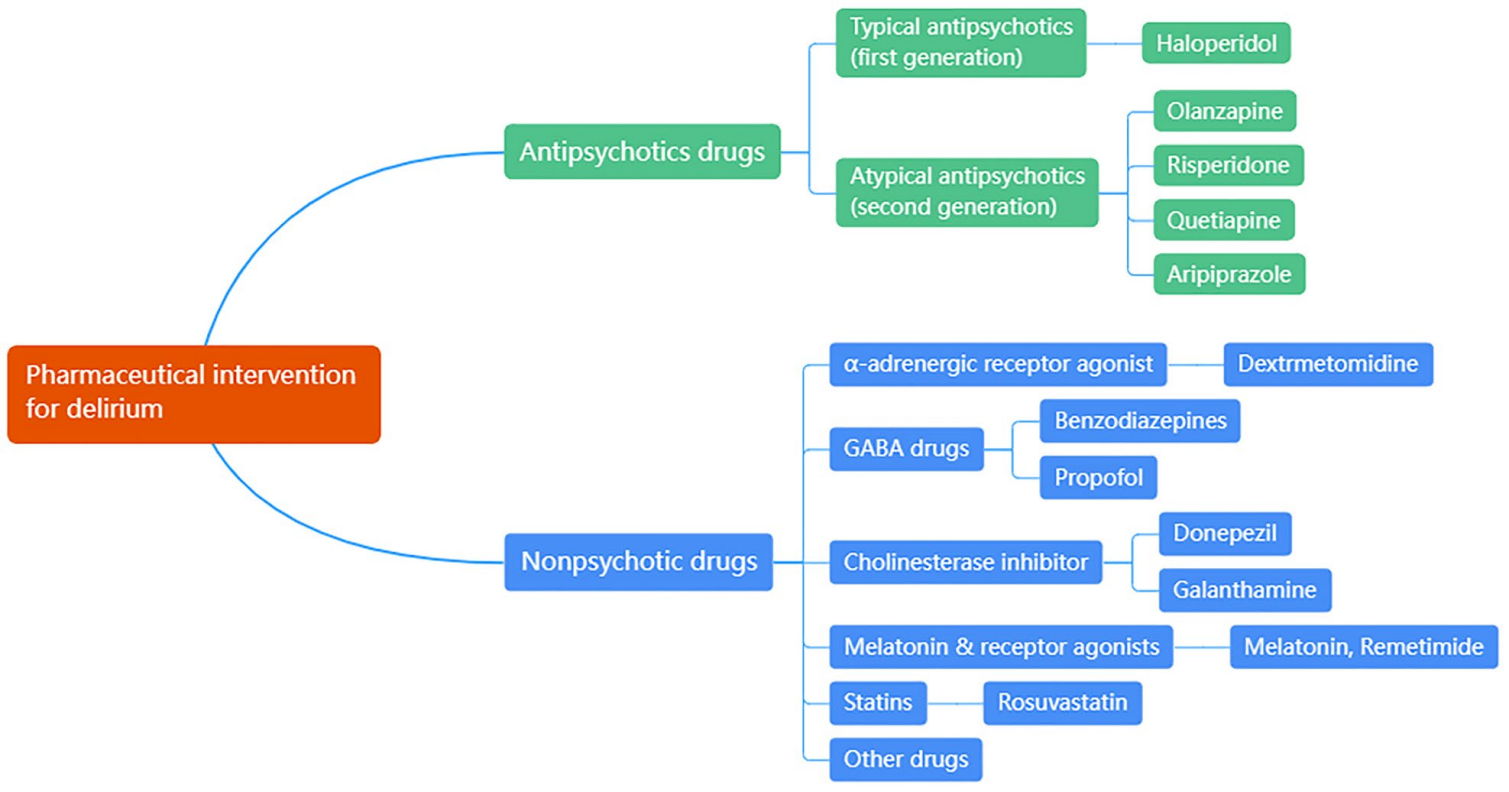
Classification of pharmaceutical interventions for delirium.

Drugs used for delirium prevention and treatment are mainly divided into antipsychotic drugs and nonpsychotic drugs (Figure 2). Antipsychotics have been reported to improve clinical symptoms, shorten duration, and reduce the severity of delirium [75]. However, antipsychotics such as haloperidol and ziprasidone have not been shown to reduce the rate of delirium occurrence [72,76,77] or shorten the duration of ICU delirium, and their high incidence of adverse reactions has also been criticized, as confirmed in recent years, by numerous large-scale placebo-controlled RCTs and meta-analyses [78–81]. For nonpsychotic drugs, the use dexmedetomidine was related with reduced risk of ICU delirium [72,73,82], duration of mechanical ventilation and ICU length of stay comparing to other sedatives, due to its low risk of respiratory depression [73]. Among atypical antipsychotic drugs, ICU patients with delirium who received quetiapine had a reduced duration of delirium [83]. A recent meta-analysis showed that olanzapine did not have a clear beneficial effect compared with other delirium drugs [84]. At present, few scholars have studied the safety of delirium drugs in nonmechanically ventilated ICU populations [3]. This field requires further exploration.

Several studies have developed and tested other delirium drugs with potential therapeutic targets. The application of exogenous melatonin and melatonin receptor agonists such as ramelteon was associated with improved sleep, reduced the incidence of delirium, and shortened period of ventilators [85–87]. The inflammatory response is another important target of current research, and critically ill patients often have complications such as organ infection or systemic inflammatory responses. Patients with delirium have been reported to benefit from statins, probably through their anti-inflammatory effects [88–90]. Further high-quality studies focused on delirium prevention and treatment are still needed.

## 5. Summary and outlook

Delirium is a state of abnormal brain function due mostly to pathological or functional changes in the brain parenchyma. Different patients may face different contributing factors, such as sleep deprivation and abnormal sleep rhythm caused by continuous ECG monitoring; light stimulation and noise; the application of hormones, anaesthetics and cholinergic drugs; ischemia; hypoxia; and cerebrovascular disease. Early assessment of risk factors in high-risk patients to predict the occurrence of delirium and avoid the contributions of known factors is an ideal management strategy for such patients. There is currently no good method for predicting and preventing delirium. Whether delirium can be predicted and how to develop a good prediction and prevention method will continue to challenge intensivists and neurologists. Further research into delirium treatment drugs and neurocritical care practice strategies is warranted to determine both their efficacy and safety.

## Data Availability

Data availability is not applicable to this article, as no new data were created or analyzed in this study.

## References

[1] Salluh JI, Soares M, Teles JM, et al. Delirium epidemiology in critical care (DECCA): an international study. Crit Care. 2010;14(6):R210. doi: 10.1186/cc9333. 21092264 PMC3220001

[2] Mesa P, Previgliano IJ, Altez S, et al. Delirium in a Latin American intensive care unit. A prospective cohort study of mechanically ventilated patients. Rev Bras Ter Intensiva. 2017;29:337–345. 29044304 10.5935/0103-507X.20170058 PMC5632977

[3] Liu S, Zhao R, Yang R, et al. Are dexmedetomidine and olanzapine suitable to control delirium in critically ill elderly patients? A retrospective cohort study. Biomed Pharmacother. 2021;139:111617. doi: 10.1016/j.biopha.2021.111617. 33915500

[4] Powers WJ, Rabinstein AA, Ackerson T, et al. Guidelines for the early management of patients with acute ischemic stroke: 2019 update to the 2018 guidelines for the early management of acute ischemic stroke: a guideline for healthcare professionals from the American Heart Association/American Stroke Association. Stroke. 2019;50(12):e344–e418. doi: 10.1161/STR.0000000000000211. 31662037

[5] Devlin JW, Skrobik Y, Gélinas C, et al. Clinical practice guidelines for the prevention and management of pain, agitation/sedation, delirium, immobility, and sleep disruption in adult patients in the ICU. Crit Care Med. 2018;46(9):e825–e873. doi: 10.1097/CCM.0000000000003299. 30113379

[6] Genoni F, Guerrini M, Sannino P, et al. Italian pediatric intensive care units need to improve the assessment of delirium, like many other Countries. Minerva Anestesiol. 2023;89(11):1060–1061. doi: 10.23736/S0375-9393.23.17452-9. 37272275

[7] Morandi A, Piva S, Ely EW, et al. Worldwide survey of the "assessing pain, both spontaneous awakening and breathing trials, choice of drugs, delirium monitoring/management, early exercise/mobility, and family empowerment" (ABCDEF) bundle. Crit Care Med. 2017;45(11):e1111–e1122. doi: 10.1097/CCM.0000000000002640. 28787293 PMC5640479

[8] Galazzi A, Giusti GD, Pagnucci N, et al. Assessment of delirium in adult patients in Intensive Care Unit: Italian critical care nurses best practices. Intensive Crit Care Nurs. 2021;66:103072. doi: 10.1016/j.iccn.2021.103072. 34059415

[9] Ely EW, Inouye SK, Bernard GR, et al. Delirium in ­mechanically ventilated patients: validity and reliability of the confusion assessment method for the intensive care unit (CAM-ICU). JAMA. 2001;286(21):2703–2710. doi: 10.1001/jama.286.21.2703. 11730446

[10] Gusmao-Flores D, Salluh JI, Chalhub R, et al. The confusion assessment method for the intensive care unit (CAM-ICU) and intensive care delirium screening checklist (ICDSC) for the diagnosis of delirium: a systematic review and meta-analysis of clinical studies. Crit Care. 2012;16(4):R115. doi: 10.1186/cc11407. 22759376 PMC3580690

[11] Bergeron N, Dubois MJ, Dumont M, et al. Intensive care delirium screening checklist: evaluation of a new screening tool. Intensive Care Med. 2001;27(5):859–864. doi: 10.1007/s001340100909. 11430542

[12] Smith HAB, Besunder JB, Betters KA, et al. 2022 society of critical care medicine clinical practice guidelines on prevention and management of pain, agitation, neuromuscular blockade, and delirium in critically ill pediatric patients with consideration of the ICU environment and early mobility. Pediatr Crit Care Med. 2022;23(2):e74–e110. doi: 10.1097/PCC.0000000000002873. 35119438

[13] Song R, Song G, Xie P, et al. [Diffusion tensor imaging and resting-state functional magnetic resonance imaging in patients with delirium in intensive care unit]. Zhonghua Wei Zhong Bing Ji Jiu Yi Xue. 2020;32(1):88–93. doi: 10.3760/cma.j.cn121430-20190905-00016. 32148238

[14] Haggstrom L, Welschinger R, Caplan GA. Functional neuroimaging offers insights into delirium pathophysiology: a systematic review. Australas J Ageing. 2017;36(3):186–192. doi: 10.1111/ajag.12417. 28519903

[15] Orhun G, Esen F, Özcan PE, et al. neuroimaging findings in sepsis-induced brain dysfunction: association with clinical and laboratory findings. Neurocrit Care. 2019;30(1):106–117. doi: 10.1007/s12028-018-0581-1. 30027347

[16] Boukrina O, Kowalczyk M, Koush Y, et al. Brain network dysfunction in poststroke delirium and spatial neglect: an fMRI study. Stroke. 2022;53(3):930–938. doi: 10.1161/STROKEAHA.121.035733. 34619987 PMC8885764

[17] Schreiber MP, Colantuoni E, Bienvenu OJ, et al. Corticosteroids and transition to delirium in patients with acute lung injury. Crit Care Med. 2014;42(6):1480–1486. doi: 10.1097/CCM.0000000000000247. 24589640 PMC4028387

[18] Pun BT, Badenes R, Heras La Calle G, et al. Prevalence and risk factors for delirium in critically ill patients with COVID-19 (COVID-D): a multicentre cohort study. Lancet Respir Med. 2021;9(3):239–250. doi: 10.1016/S2213-2600(20)30552-X. 33428871 PMC7832119

[19] Chan CK, Song Y, Greene R, et al. Meta-analysis of ICU delirium biomarkers and their alignment with the NIA-AA research framework. Am J Crit Care. 2021;30(4):312–319. doi: 10.4037/ajcc2021771. 34195769 PMC8897570

[20] Lei W, Ren Z, Su J, et al. Immunological risk factors for sepsis-associated delirium and mortality in ICU patients. Front Immunol. 2022;13:940779. doi: 10.3389/fimmu.2022.940779. 36203605 PMC9531264

[21] Wang J, Ji Y, Wang N, et al. Risk factors for the incidence of delirium in cerebrovascular patients in a neurosurgery intensive care unit: a prospective study. J Clin Nurs. 2018;27(1–2):407–415. doi: 10.1111/jocn.13943. 28677160

[22] Li X, Zhang L, Gong F, et al. Incidence and risk factors for delirium in older patients following intensive care unit ­admission: a prospective observational study. J Nurs Res. 2020;28(4):e101. doi: 10.1097/jnr.0000000000000384. 32692119

[23] Hshieh TT, Fong TG, Marcantonio ER, et al. Cholinergic deficiency hypothesis in delirium: a synthesis of current evidence. J Gerontol A Biol Sci Med Sci. 2008;63(7):764–772. doi: 10.1093/gerona/63.7.764. 18693233 PMC2917793

[24] Hughes CG, Boncyk CS, Fedeles B, et al. Association ­between cholinesterase activity and critical illness brain dysfunction. Crit Care. 2022;26(1):377. doi: 10.1186/s13054-022-04260-1. 36474266 PMC9724294

[25] Zujalovic B, Mayer B, Hafner S, et al. AChE-activity in critically ill patients with suspected septic encephalopathy: a prospective, single-centre study. BMC Anesthesiol. 2020;20(1):287. doi: 10.1186/s12871-020-01204-6. 33203376 PMC7670732

[26] Yoshitaka S, Egi M, Kanazawa T, et al. The association of plasma gamma-aminobutyric acid concentration with postoperative delirium in critically ill patients. Crit Care Resusc. 2014;16(4):269–273. doi: 10.1016/S1441-2772(23)01618-6. 25437220

[27] Cortese GP, Burger C. Neuroinflammatory challenges compromise neuronal function in the aging brain: postoperative cognitive delirium and Alzheimer’s disease. Behav Brain Res. 2017;322(Pt B):269–279. doi: 10.1016/j.bbr.2016.08.027. 27544872 PMC5450823

[28] Neerland BE, Hall RJ, Seljeflot I, et al. Associations ­between delirium and preoperative cerebrospinal fluid C-reactive protein, interleukin-6, and interleukin-6 receptor in individuals with acute hip fracture. J Am Geriatr Soc. 2016;64(7):1456–1463. doi: 10.1111/jgs.14238. 27341529

[29] Kealy J, Murray C, Griffin EW, et al. Acute inflammation alters brain energy metabolism in mice and humans: role in suppressed spontaneous activity, impaired cognition, and delirium. J Neurosci. 2020;40(29):5681–5696. doi: 10.1523/JNEUROSCI.2876-19.2020. 32513828 PMC7363463

[30] Egberts A, Mattace-Raso FU. Increased neutrophil-lymphocyte ratio in delirium: a pilot study. Clin Interv Aging. 2017;12:1115–1121. doi: 10.2147/CIA.S137182. 28769556 PMC5529095

[31] Khan BA, Perkins AJ, Prasad NK, et al. Biomarkers of ­delirium duration and delirium severity in the ICU. Crit Care Med. 2020;48(3):353–361. doi: 10.1097/CCM.0000000000004139. 31770149 PMC7242000

[32] Smith RJ, Lachner C, Singh VP, et al. Cytokine profiles in intensive care unit delirium. Acute Crit Care. 2022;37(3):415–428. doi: 10.4266/acc.2021.01508. 35791660 PMC9475146

[33] Harris MA, Cox SR, Brett CE, et al. Cognitive ability across the life course and cortisol levels in older age. Neurobiol Aging. 2017;59:64–71. doi: 10.1016/j.neurobiolaging.2017.07.012. 28865298

[34] Nguyen DN, Huyghens L, Zhang H, et al. Cortisol is an associated-risk factor of brain dysfunction in patients with severe sepsis and septic shock. Biomed Res Int. 2014;2014:712742–712747. doi: 10.1155/2014/712742. 24883321 PMC4022165

[35] Krewulak KD, Stelfox HT, Leigh JP, et al. Incidence and prevalence of delirium subtypes in an adult ICU: a systematic review and meta-analysis. Crit Care Med. 2018;46(12):2029–2035. doi: 10.1097/CCM.0000000000003402. 30234569

[36] Inouye SK, van Dyck CH, Alessi CA, et al. Clarifying confusion: the confusion assessment method. A new method for detection of delirium. Ann Intern Med. 1990;113(12):941–948. doi: 10.7326/0003-4819-113-12-941. 2240918

[37] Arbanas G. Diagnostic and statistical manual of mental disorders (DSM-5). Codas. 2015;25:591–644. 10.1590/s2317-17822013000200017 24413388

[38] World Health Organization. The ICD-10 classification of mental and behavioural disorders: clinical descriptions and diagnostic guidelines[J]. World Health Organization, 1992, 362.

[39] Breitbart W, Rosenfeld B, Roth A, et al. The memorial delirium assessment scale. J Pain Symptom Manage. 1997;13(3):128–137. doi: 10.1016/s0885-3924(96)00316-8. 9114631

[40] Psychiatric CSo. CCMD-3 classification and diagnostic criteria of mental disorders in China. Shandong Science and Technology Press. 2001.

[41] Trzepacz PT, Mittal D, Torres R, et al. Validation of the delirium rating scale-revised-98: comparison with the delirium rating scale and the cognitive test for delirium. J Neuropsychiatry Clin Neurosci. 2001;13(2):229–242. doi: 10.1176/jnp.13.2.229. 11449030

[42] Inouye SK, Kosar CM, Tommet D, et al. The CAM-S: ­development and validation of a new scoring system for delirium severity in 2 cohorts. Ann Intern Med. 2014;160(8):526–533. doi: 10.7326/M13-1927. 24733193 PMC4038434

[43] Hart RP, Levenson JL, Sessler CN, et al. Validation of a cognitive test for delirium in medical ICU patients. Psychosomatics. 1996;37(6):533–546. doi: 10.1016/S0033-3182(96)71517-7. 8942204

[44] MacLullich AM, Shenkin SD. The 4 ‘A’s test for detecting delirium in acute medical patients: a diagnostic accuracy study. Health Technol Assess. 2019;23:1–194. 10.3310/hta23400 PMC6709509 31397263

[45] Sachdev PS, Blacker D, Blazer DG, et al. Classifying neurocognitive disorders: the DSM-5 approach. Nat Rev Neurol. 2014;10(11):634–642. doi: 10.1038/nrneurol.2014.181. 25266297

[46] Gross AL, Tommet D, D’Aquila M, et al. Harmonization of delirium severity instruments: a comparison of the DRS-R-98, MDAS, and CAM-S using item response theory. BMC Med Res Methodol. 2018;18(1):92. doi: 10.1186/s12874-018-0552-4. 30200896 PMC6131747

[47] De J, Wand AP. Delirium screening: a systematic review of delirium screening tools in hospitalized patients. Gerontologist. 2015;55(6):1079–1099. doi: 10.1093/geront/gnv100. 26543179

[48] Chen TJ, Chung YW, Chang HR, et al. Diagnostic accuracy of the CAM-ICU and ICDSC in detecting intensive care unit delirium: a bivariate meta-analysis. Int J Nurs Stud. 2021;113:103782. doi: 10.1016/j.ijnurstu.2020.103782. 33120134

[49] Ewers R, Bloomer MJ, Hutchinson A. An exploration of the reliability and usability of two delirium screening tools in an Australian intensive care unit: a pilot study. Intensive Crit Care Nurs. 2021;62:102919. doi: 10.1016/j.iccn.2020.102919. 32873426

[50] Rosa RG, Falavigna M, da Silva DB, et al. Effect of flexible family visitation on delirium among patients in the intensive care unit: the ICU visits randomized clinical trial. JAMA. 2019;322(3):216–228. doi: 10.1001/jama.2019.8766. 31310297 PMC6635909

[51] Wibrow B, Martinez FE, Myers E, et al. Prophylactic melatonin for delirium in intensive care (Pro-MEDIC): a randomized controlled trial. Intensive Care Med. 2022;48(4):414–425. doi: 10.1007/s00134-022-06638-9. 35220473

[52] Khan BA, Perkins AJ, Campbell NL, et al. Pharmacological management of delirium in the intensive care unit: a randomized pragmatic clinical trial. J Am Geriatr Soc. 2019;67(5):1057–1065. doi: 10.1111/jgs.15781. 30681720 PMC6492267

[53] Barr J, Fraser GL, Puntillo K, et al. Clinical practice guidelines for the management of pain, agitation, and delirium in adult patients in the intensive care unit. Crit Care Med. 2013;41(1):263–306. doi: 10.1097/CCM.0b013e3182783b72. 23269131

[54] Ely EW, Truman B, Shintani A, et al. Monitoring sedation status over time in ICU patients: reliability and validity of the Richmond Agitation-Sedation Scale (RASS). JAMA. 2003;289(22):2983–2991. doi: 10.1001/jama.289.22.2983. 12799407

[55] Hughes CG, Mailloux PT, Devlin JW, et al. Dexmedetomidine or propofol for sedation in mechanically ventilated adults with sepsis. N Engl J Med. 2021;384(15):1424–1436. doi: 10.1056/NEJMoa2024922. 33528922 PMC8162695

[56] Dallı ÖE, Yıldırım Y, Aykar F, et al. The effect of music on delirium, pain, sedation and anxiety in patients receiving mechanical ventilation in the intensive care unit. Intensive Crit Care Nurs. 2023;75:103348. doi: 10.1016/j.iccn.2022.103348. 36470699

[57] Hui D, De La Rosa A, Wilson A, et al. Neuroleptic strategies for terminal agitation in patients with cancer and delirium at an acute palliative care unit: a single-centre, double-blind, parallel-group, randomised trial. Lancet Oncol. 2020;21(7):989–998. doi: 10.1016/S1470-2045(20)30307-7. 32479786 PMC7433183

[58] Han Y, Zhang W, Liu J, et al. Metabolomic and lipidomic profiling of preoperative CSF in elderly hip fracture patients with postoperative delirium. Front Aging Neurosci. 2020;12:570210. doi: 10.3389/fnagi.2020.570210. 33192460 PMC7642614

[59] Tripp BA, Dillon ST, Yuan M, et al. Targeted metabolomics analysis of postoperative delirium. Sci Rep. 2021;11(1):1521. doi: 10.1038/s41598-020-80412-z. 33452279 PMC7810737

[60] Erstad BL, Patanwala AE. Ketamine for analgosedation in critically ill patients. J Crit Care. 2016;35:145–149. doi: 10.1016/j.jcrc.2016.05.016. 27481750

[61] Gile J, Scott B, Eckle T. The period 2 enhancer nobiletin as novel therapy in murine models of circadian disruption resembling delirium. Crit Care Med. 2018;46(6):e600–e608. doi: 10.1097/CCM.0000000000003077. 29489460 PMC5953804

[62] Han JH, Chen A, Vasilevskis EE, et al. Supratherapeutic psychotropic drug levels in the emergency department and their association with delirium duration: a preliminary study. J Am Geriatr Soc. 2019;67(11):2387–2392. doi: 10.1111/jgs.16156. 31503339 PMC7029781

[63] Lu Y, Chen L, Ye J, et al. Surgery/Anesthesia disturbs mitochondrial fission/fusion dynamics in the brain of aged mice with postoperative delirium. Aging (Albany NY). 2020;12(1):844–865. doi: 10.18632/aging.102659. 31929114 PMC6977661

[64] Enomoto R, Lee-Hiraiwa E. Elimination of the causes of poor sleep underlying delirium is a basic strategy to prevent delirium. Curr Mol Pharmacol. 2021;14(2):132–137. doi: 10.2174/1874467213666200424150709. 32329703

[65] Baron R, Binder A, Biniek R, et al. Evidence and consensus based guideline for the management of delirium, analgesia, and sedation in intensive care medicine. Revision 2015 (DAS-Guideline 2015) - short version. Ger Med Sci. 2015;13:doc19. doi: 10.3205/000223. 26609286 PMC4645746

[66] Vincent JL, Shehabi Y, Walsh TS, et al. Comfort and patient-centred care without excessive sedation: the eCASH concept. Intensive Care Med. 2016;42(6):962–971. doi: 10.1007/s00134-016-4297-4. 27075762 PMC4846689

[67] Godfrey M, Green J, Smith J, et al. Process of implementing and delivering the Prevention of Delirium system of care: a mixed method preliminary study. BMC Geriatr. 2019;20(1):1. doi: 10.1186/s12877-019-1374-x. 31892317 PMC6938603

[68] Kyeong S, Choi SH, Eun Shin J, et al. Functional connectivity of the circadian clock and neural substrates of sleep-wake disturbance in delirium. Psychiatry Res Neuroimaging. 2017;264:10–12. doi: 10.1016/j.pscychresns.2017.03.017. 28390292

[69] Wang S, Hammes J, Khan S, et al. Improving recovery and outcomes every day after the ICU (IMPROVE): study protocol for a randomized controlled trial. Trials. 2018;19(1):196. doi: 10.1186/s13063-018-2569-8. 29580264 PMC5869765

[70] Khan SH, Xu C, Purpura R, et al. Decreasing delirium through music: a randomized pilot trial. Am J Crit Care. 2020;29(2):e31–e38. doi: 10.4037/ajcc2020175. 32114612 PMC7666845

[71] Hayhurst CJ, Farrin E, Hughes CG. The effect of ketamine on delirium and opioid-induced hyperalgesia in the Intensive Care Unit. Anaesth Crit Care Pain Med. 2018;37(6):525–527. doi: 10.1016/j.accpm.2018.11.001. 30573208

[72] Burry LD, Cheng W, Williamson DR, et al. Pharmacological and non-pharmacological interventions to prevent delirium in critically ill patients: a systematic review and network meta-analysis. Intensive Care Med. 2021;47(9):943–960. doi: 10.1007/s00134-021-06490-3. 34379152 PMC8356549

[73] Lewis K, Alshamsi F, Carayannopoulos KL, et al. Dexmedetomidine vs other sedatives in critically ill mechanically ventilated adults: a systematic review and meta-analysis of randomized trials. Intensive Care Med. 2022;48(7):811–840. doi: 10.1007/s00134-022-06712-2. 35648198

[74] Mody K, Kaur S, Mauer EA, et al. Benzodiazepines and development of delirium in critically ill children: estimating the causal effect. Crit Care Med. 2018;46(9):1486–1491. doi: 10.1097/CCM.0000000000003194. 29727363 PMC6095819

[75] Burry L, Mehta S, Perreault MM, et al. Antipsychotics for treatment of delirium in hospitalised non-ICU patients. Cochrane Database Syst Rev. 2018;6(6):Cd005594. doi: 10.1002/14651858.CD005594.pub3. 29920656 PMC6513380

[76] Neufeld KJ, Yue J, Robinson TN, et al. Antipsychotic medication for prevention and treatment of delirium in hospitalized adults: a systematic review and meta-analysis. J Am Geriatr Soc. 2016;64(4):705–714. doi: 10.1111/jgs.14076. 27004732 PMC4840067

[77] Marra A, Vargas M, Buonanno P, et al. Haloperidol for preventing delirium in ICU patients: a systematic review and meta-analysis. Eur Rev Med Pharmacol Sci. 2021;25(3):1582–1591. doi: 10.26355/eurrev_202102_24868. 33629327

[78] Andersen-Ranberg NC, Poulsen LM, Perner A, AID-ICU Trial Group, et al. Haloperidol for the Treatment of Delirium in ICU Patients. N Engl J Med. 2022;387(26):2425–2435. doi: 10.1056/NEJMoa2211868. 36286254

[79] Zayed Y, Barbarawi M, Kheiri B, et al. Haloperidol for the management of delirium in adult intensive care unit patients: A systematic review and meta-analysis of randomized controlled trials. J Crit Care. 2019;50:280–286. doi: 10.1016/j.jcrc.2019.01.009. 30665181

[80] van den Boogaard M, Slooter AJC, Brüggemann RJM, et al. Effect of haloperidol on survival among critically ill adults with a high risk of delirium: the REDUCE randomized clinical trial. JAMA. 2018;319(7):680–690. doi: 10.1001/jama.2018.0160. 29466591 PMC5839284

[81] Girard TD, Exline MC, Carson SS, et al. Haloperidol and ziprasidone for treatment of delirium in critical illness. N Engl J Med. 2018;379(26):2506–2516. doi: 10.1056/NEJMoa1808217. 30346242 PMC6364999

[82] Herling SF, Greve IE, Vasilevskis EE, et al. Interventions for preventing intensive care unit delirium in adults. Cochrane Database Syst Rev. 2018;11(11):Cd009783. doi: 10.1002/14651858.CD009783.pub2. 30484283 PMC6373634

[83] Devlin JW, Roberts RJ, Fong JJ, et al. Efficacy and safety of quetiapine in critically ill patients with delirium: a prospective, multicenter, randomized, double-blind, placebo-controlled pilot study. Crit Care Med. 2010;38(2):419–427. doi: 10.1097/CCM.0b013e3181b9e302. 19915454

[84] Liu SB, Liu S, Gao K, et al. Olanzapine for the treatment of ICU delirium: a systematic review and meta-analysis. Ther Adv Psychopharmacol. 2023;13:20451253231152113. doi: 10.1177/20451253231152113. 36845642 PMC9944192

[85] Campbell AM, Axon DR, Martin JR, et al. Melatonin for the prevention of postoperative delirium in older adults: a systematic review and meta-analysis. BMC Geriatr. 2019;19(1):272. doi: 10.1186/s12877-019-1297-6. 31619178 PMC6796479

[86] Zhang Q, Gao F, Zhang S, et al. Prophylactic use of exogenous melatonin and melatonin receptor agonists to improve sleep and delirium in the intensive care units: a systematic review and meta-analysis of randomized controlled trials. Sleep Breath. 2019;23(4):1059–1070. doi: 10.1007/s11325-019-01831-5. 31119597

[87] Jaiswal SJ, McCarthy TJ, Wineinger NE, et al. Melatonin and sleep in preventing hospitalized delirium: a randomized clinical trial. Am J Med. 2018;131(9):1110–1117.e1114. doi: 10.1016/j.amjmed.2018.04.009. 29729237 PMC6163056

[88] Page VJ, Casarin A, Ely EW, et al. Evaluation of early ­administration of simvastatin in the prevention and treatment of delirium in critically ill patients undergoing ­mechanical ventilation (MoDUS): a randomised, double-blind, placebo-controlled trial. Lancet Respir Med. 2017;5(9):727–737. doi: 10.1016/S2213-2600(17)30234-5. 28734823

[89] Agus A, Phair G, Page VJ, et al. Simvastatin for the prevention and treatment of delirium in critically ill, mechanically ventilated patients (MoDUS): a cost-effectiveness analysis. Lancet Respir Med. 2018;6(3):e9–e10. doi: 10.1016/S2213-2600(18)30070-5. 29508709

[90] Needham DM, Colantuoni E, Dinglas VD, et al. Rosuvastatin versus placebo for delirium in intensive care and subsequent cognitive impairment in patients with sepsis-associated acute respiratory distress syndrome: an ancillary study to a randomised controlled trial. Lancet Respir Med. 2016;4(3):203–212. doi: 10.1016/S2213-2600(16)00005-9. 26832963 PMC4792772

